# From procurement to disposal: a framework for an activity-based cost analysis of reusable and disposable trocars in laparoscopic cholecystectomy

**DOI:** 10.1007/s00464-026-12827-0

**Published:** 2026-04-21

**Authors:** Rhiannon C. Reising, Brigitte A. B. Essers, Myrthe M. M. Eussen, Rebekka M. Brandwijk, Bradley Sugden, Nicole D. Bouvy, Manuela Joore, Merel L. Kimman

**Affiliations:** 1 Department of Clinical Epidemiology and Medical Technology Assessment, Maastricht University Medical Centre, Maastricht, The Netherlands; 2 Department of Health Services Research, Care and Public Health Research Institute (CAPHRI), Maastricht University, Maastricht, The Netherlands; 3 Department of Surgery, Maastricht University Medical Centre, Maastricht, The Netherlands; 4 NUTRIM School of Nutrition and Translational Research in Metabolism, Maastricht University, Maastricht, The Netherlands; 5 Aachen-Maastricht Institute for Biobased Materials (AMIBM), Maastricht University, Geleen, The Netherlands; 6 Department of Surgery, Leiden University Medical Centre, Leiden, The Netherlands

**Keywords:** Laparoscopic cholecystectomy, Environmental sustainability, Activity-based costing, Cost analysis, Trocars

## Abstract

**Background:**

Operating rooms are responsible for a substantial share of hospital waste, with laparoscopic procedures being particularly resource-intensive due to their reliance on disposable instruments. In a laparoscopic cholecystectomy (LC), disposable products account for approximately 40% of emissions, with trocars being key contributors. Reusable trocars lower environmental impact and evidence suggests that costs can be reduced. Accordingly, this study compares the costs of reusable versus disposable trocars during LC in a Dutch academic hospital and develops an adaptable framework for cost evaluation in other healthcare settings.

**Methods:**

An activity-based costing analysis was conducted to estimate the per-procedure costs (i.e. per LC), including the costs for acquisition, sterilisation, storage, and waste disposal. This was based on an LC using four trocars: two 5 mm and two 12 mm trocars. Resource use was based on expert input, and unit costs were obtained from hospital procurement data. The costing framework was designed to be adaptable to different institutional contexts. Uncertainty in the cost difference was assessed using probabilistic sensitivity analysis with 1000 Monte Carlo simulations, alongside one-way, two-way, and scenario analyses.

**Results:**

Acquisition costs per LC were substantially lower for reusable trocars (€8.15) than for disposable trocars (€89.55). Although reusable trocars incurred sterilisation costs (€13.31 per procedure), total costs, including all activities involved in LC, remained lower (€683.00 vs €751.43), resulting in savings of €68.80 per procedure. Reusable trocars were cost-saving in all sensitivity analyses. Estimated annual savings were €10,264 at institutional level (based on 150 procedures) and €1.54 million nationally (based on 22,500 procedures).

**Conclusion:**

Reusable trocars are cost-saving compared to disposable trocars in LC. The adaptable activity-based costing framework developed in this study enables healthcare institutions to evaluate the economic impact of reusable versus disposable surgical instruments within their own settings, supporting both cost reduction and environmental sustainability.

**Supplementary Information:**

The online version contains supplementary material available at 10.1007/s00464-026-12827-0.

The healthcare sector plays a crucial role in planetary health not only by protecting human health but also by contributing to environmental impacts such as air and water pollution, ecosystem degradation, and biodiversity loss [1, 2]. In response to these challenges, the World Health Organisation (WHO) has called for action to develop environmentally sustainable and climate-resilient healthcare systems [3]. The adoption of reusable instruments within healthcare, as compared with disposable/single-use instruments, can offer environmental benefits [4–8]. Furthermore, there is growing evidence to suggest that such adoption may offer economic benefits. This is increasingly relevant in the context of rising healthcare costs and resource constraints.

In the Netherlands, the healthcare sector accounts for 4–8% of the national carbon footprint [9]. Moreover, it is responsible for 13% of material extraction and 4% of direct and indirect waste generation [10, 11]. Within healthcare, the operating room is disproportionally resource-intensive, producing 20–33% of total hospital waste [12, 13].

To mitigate these impacts, researchers and policy makers have proposed embedding circular economy principles into healthcare systems. A circular economy aims to eliminate waste and regenerate natural systems by maintaining materials in use through strategies such as maintenance, reuse, refurbishment, remanufacturing, recycling, and composting [14]. The 10-R framework of circular strategies conceptualises the circular economy. This includes (0) Refuse, (1) Rethink, (2) Reduce, (3) Reuse, (4) Repair, (5) Refurbish, (6) Remanufacture, (7) Repurpose, (8) Recycle, and (9) Recover [15].

Within the surgical discipline, laparoscopic and robotic procedures are particularly emission-intensive due to their heavy reliance on disposable instruments and materials [16]. This is exemplified by laparoscopic cholecystectomy (LC), where a substantial portion of emissions, 40%, stems from disposable products [17]. Among these, trocars are key contributors. These instruments, used in every laparoscopic surgery, are often disposable and discarded after one procedure. In the Netherlands alone, an estimated 90,000 trocars are used annually for LCs, contributing substantially to surgical waste [18]. Adopting reusable trocars aligns with a circular economy offering a promising opportunity for waste reduction.

Additionally adopting reusable instruments can reduce costs as seen in previous research. A systematic review by Chauvet et al. [19] found that reusable laparoscopic instruments, including trocars, scissors, and staplers, were consistently less expensive than their disposable counterparts. Similarly, Eussen et al. [7] reviewed 20 studies and reported a general cost advantage for reusable instruments. However, the cost analyses in these studies varied in scope. While most included purchase price, fewer considered maintenance, sterilisation, or disposal costs.

To our knowledge, no study has provided a comprehensive cost analysis that accounts for all relevant components of trocar use within the hospital setting including storage, reprocessing, and waste disposal. Given the growing focus on environmentally sustainable surgical practices, a detailed economic comparison of reusable versus disposable trocars is therefore warranted.

This study seeks to address this gap by evaluating the costs associated within the hospital context, with a specific focus on LC. In addition, we present a framework that can be populated with institution-specific operational data, enabling healthcare organisations to conduct context-appropriate assessments of the economic implications of adopting reusable surgical instruments for an LC.

## Methods

### Study design

We conducted this study in the Netherlands at Maastricht University Medical Centre (MUMC +). To compare the costs of reusable and disposable trocars in an LC, we developed a costing framework based on an activity-based costing (ABC) approach. This is a bottom-up approach, where one collects detailed data about each relevant activity. ABC typically follows three standard steps: mapping activities, calculating the cost of each activity, and aggregating these to calculate the total cost of each procedure [20].

This research was carried out as part of the Creating A healthieR Environment for FutuRE patiEnts (CAREFREE) project. This project aims to identify key sources of pollution within the operating room and change behaviour among stakeholders. It identifies existing knowledge gaps and develops practical tools designed to foster trust, encourage acceptance, and facilitate behavioural change across the wide range of stakeholders involved. The project adopts a multidisciplinary approach, involving key stakeholders including researchers, clinicians, and industry professionals from fields spanning social psychology, philosophy, sustainability, surgery, anaesthesiology, pharmacology and toxicology, health economics, and operations research.

## Mapping activities

Reusable and disposable trocars were followed throughout their respective journeys in the hospital, covering all activities involved in their use for LC. Two distinct process maps were developed through an iterative, feedback-driven approach informed by relevant literature, standard operating procedures, and information provided by key stakeholders in MUMC + . Stakeholders included one expert from the sterilisation department, one expert from the internal distribution centre, one expert from the internal logistics department, one expert from the medical technology department, and one expert from the energy department. The process maps were subsequently presented at a CAREFREE consortium meeting, where the participants validated each step to ensure completeness and to confirm that no critical elements were overlooked.

**Fig. 1:**
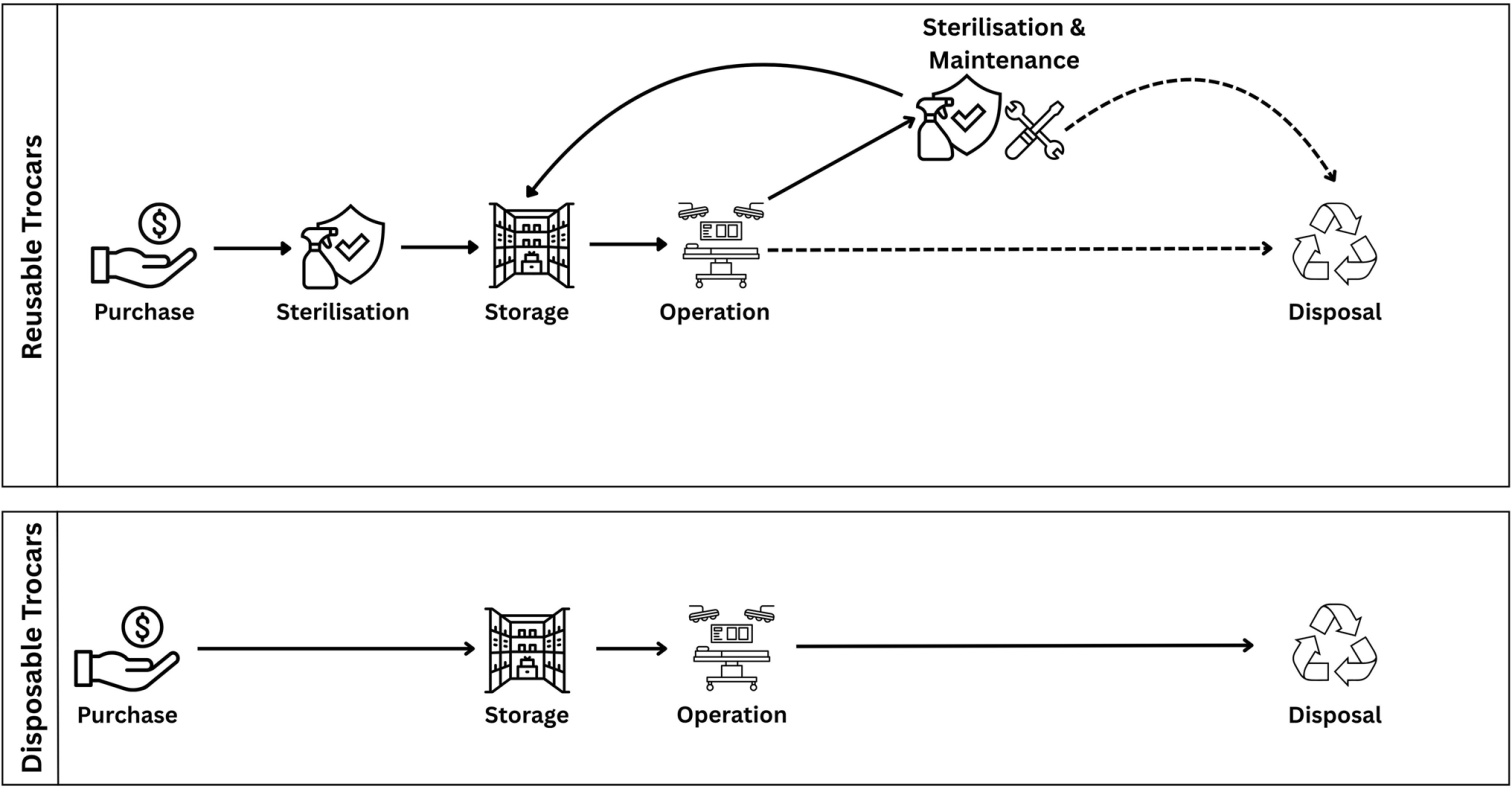
Process maps of reusable and disposable trocars

For reusable trocars, the process map included the following steps: purchase, sterilisation, storage, surgical operation, and post-operative pathways, which involved either returning to the cleaning and sterilisation department and storage, undergoing maintenance, or being disposed if deemed no longer usable. For disposable trocars, the process was linear, consisting of purchase, storage, surgical operation, and disposal (see Fig. 1).

## Identification and measurement of cost parameters for each activity

Along the process maps, all resource use and cost prices per unit were identified to determine the cost per procedure for the reusable and the disposable trocars.

### Purchasing

For the LC procedure at MUMC + four trocars are required. Two 5 mm trocars and 12 mm trocars are needed, irrespective of whether reusable or disposable trocars are used. For reusable trocars, manufacturer list prices were used to inform purchase prices. The acquisition cost for a set of reusable trocars is 2248€. To determine a purchase price per procedure, the acquisition cost was amortised over 150 uses. This estimate is based on literature and is confirmed by members of the CAREFREE consortium [21]. Reusable trocars also consist of small rubber parts that can be replaced. These include silicon valves, sealing caps, and cross ventils. The assumption is that these can be used 90 times. For the disposable trocars, the acquisition costs for a set of trocars are 89.43€. Disposable trocars can only be used once.

### Sterilisation and maintenance

For reusable trocars only, cleaning, sterilisation and maintenance were divided into four categories: materials, equipment, utilities, and personnel. Material costs included consumables such as detergents and packaging materials during the sterilisation and maintenance step. Equipment costs included the investment cost for machinery used to sterilise the trocars accounting for the depreciation, interest, maintenance, and annual number of uses [22]. The annual number of uses was obtained from the head of the sterilisation department and assumes that the machines run seven times per day during the week and three times per day on the weekend. Utility costs included water and electricity usage of the machines. For calculation purposes, it is assumed that electricity is purchased and not self-generated. Personnel costs were based on the time required for staff to complete the sterilisation process. To allocate part of the sterilisation costs per cycle of a machine to one LC procedure, we considered the capacity of the cleaning (i.e. the Thermodesinfector) and sterilisation machines (the Steriliser). The Thermodisinfector fits 12 nets each containing 8 trocars in the machine (96 trocars in total). The Steriliser fits 16 nets in one machine (128 trocars in total). The cost per unit of the items used in the sterilisation process can be seen in Appendix 1.

### Storage

Storage costs encompassed costs for storage space and personnel time for monthly stock checks. Storage costs for disposable trocars were calculated based on the space required to maintain a 10-day supply (600 trocars). The space required is 0.24 m^2^. Additionally, personnel costs were incurred for disposable trocars due to monthly stock checks. These were calculated with an hourly wage of 47€. Reusable trocars take up one-third of this space, which is 0.08m2. The total annual storage costs are based on how much it costs to build an academic hospital in Maastricht based on bouwkostenkompas.nl. These amounted to 3709€. To obtain the storage cost per procedure, the total annual storage cost was divided by the estimated 3600 laparoscopic procedures per year at MUMC + .

### Operation

The operation time including the time to place the trocars for one LC was based on MUMC + data and estimated at 60 min and assumed not to be impacted by the type of trocars used. Costs were calculated based on the cost per minute of the operating time in a conventional OR, which is 11.09€ per minute [22, 23]. This unit cost includes costs for accommodation, equipment, staff, and overheads.

### Disposal

Disposal costs were calculated by multiplying the weight of each trocar by the disposal cost per kilogram. Reusable and disposable trocars are disposed in specific hospital waste. For reusable trocars, manufacturer data on weight were used; for disposable trocars, weights were measured by the research team (RB). For reusable trocars, disposal costs were divided by 150 to obtain a per-use cost [21]. Additionally, the cost of disposing Bluewrap, which was used to pack the reusable trocars, was considered, these were disposed in the general waste. Minor components (e.g. rubbers) were excluded due to negligible weight and irregular/infrequent disposal. The cost of disposing one kilogram of trocars in the specific hospital waste is 1.23 €. The cost of disposing one kilogram of Bluewrap in the general hospital waste is 0.19€.

## Data analysis

### Base-case analysis

An activity-based cost analysis was conducted to compare reusable and disposable trocars based on a base-case scenario reflecting typical clinical practice at MUMC + . The base-case assumed a standard combination of trocars used in LC consisting of two 5 mm and two 12 mm trocars per operation. We calculated the total cost per procedure based on the process maps described in the methods.

Base-case cost estimates were derived using probabilistic analysis to account for parameter uncertainty. Mean costs per procedure and corresponding 95% credible intervals were estimated from Monte Carlo simulations conducted in Excel© with 1000 iterations, based on convergence diagnostics. Gamma distributions were assigned to all stochastic parameters. For all parameters, the standard error was assumed to be 20% of the mean. All input parameters, including corresponding standard errors and distributions, are presented in Appendix 2.

To assess the impact of the cost difference at an institutional and national scale, we multiplied the absolute total cost difference per procedure by two reference volumes: (1) the total number of LC performed at MUMC + annually (*n* = 150) and (2) the annual number of LC performed in the Netherlands (*n* = 22,500) [18].

### Sensitivity analyses

#### One-way sensitivity analysis

To assess the relative impact of individual parameters on the absolute cost difference between reusable and disposable trocars, a one-way sensitivity analysis (OWSA) was conducted. Parameters were varied one at a time, while all other inputs were kept at their base-case values. Where otherwise specified in Appendix 3, minimum and maximum values were calculated based on the assumed probability distributions, parameterised using the base-case mean and assumed standard error corresponding to the 2.5th and 97.5th distribution percentiles. The ten parameters with the greatest impact on the cost difference per procedure are shown in a tornado diagram.

#### Two-way sensitivity analysis

A two-way sensitivity analysis was performed to evaluate the combined effect of variation in the two parameters that had the greatest impact in the one-way sensitivity analysis. The results are shown in a heat diagram.

### Scenario analysis

To account for environmental externalities, we conducted a scenario analysis incorporating the social cost of carbon (SCC). Greenhouse gas emissions associated with reusable and disposable trocars were informed by published life cycle assessment data [5]. The greenhouse gas emissions for 500 functional units amount to 565 kgCO_2_eq for disposable trocars and 118 kgCO_2_eq for reusable trocars. The monetary valuation of these emissions was based on the SCC estimate reported by Rennert et al. [24], set at 185 USD per metric ton of CO_2_eq. This value was converted into euros, resulting in 158.39 €/ton CO_2_eq. Emission quantities expressed in kilograms were converted to metric tons and multiplied by the SCC to obtain the environmental cost per procedure for each trocar type.

To account for potential variability in purchasing costs, we conducted a scenario analysis using acquisition costs reported in a recent study comparing reusable and disposable trocars [4]. In that study, the median acquisition cost was €1,180 for reusable trocars and €162 for disposable trocars. As mean values were not reported, median values were used as proxies for the mean in our analysis.

## Results

### Purchase costs

The per-procedure purchase costs differed between reusable and disposable trocars. For reusable trocars, when the purchase cost was amortised over the expected 150 LC uses, the per-procedure cost was €8.15 (95% CI: 5.44–11.78). For disposable trocars, the total purchase cost of four instruments amounted to €89.55 (95% CI: 72.44–107.72) per LC.

### Sterilisation and maintenance costs

**Fig. 2:**
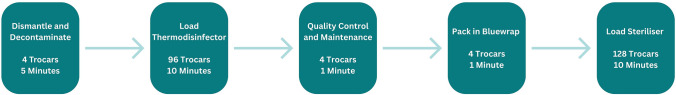
Process time estimates in the sterilisation department

Sterilisation costs for reusable trocars were calculated at €13.31 (95% CI: 8.37–20.29) per procedure. Personnel costs were €11.88 (95% CI: 6.95–18.69) per procedure and were the largest component (89.00%) of the total sterilisation costs. Personnel costs are based on processing times for the individual steps (Fig. 2). Material costs contributed €0.75 (95% CI: 0.49–1.06) per procedure (5.60%), while utility and equipment costs were minimal, at €0.04 (95% CI: 0.03–0.06) (< 1%) and €0.64 (95% CI: 0.39–0.99) (4.80%) per procedure, respectively. Detailed resource-use quantities (e.g. detergent volumes, electricity, and water consumption) are provided in Appendix 4.

### Storage costs

Reusable trocars required less storage space and no personnel input for stock checks, resulting in a total storage cost of €0.08 (95% CI: 0.04–0.13) per procedure. Disposable trocars incurred a total storage cost of €0.27 (95% CI: 0.15–0.44) per procedure, comprising €0.24 (95% CI: 0.13–0.42) for storage volume and €0.03 (95% CI: 0.01–0.04) for personnel costs related to stock checks, which were 10 min of stock checks per month.

### Operation costs

With a total operating time of 60 min, and a cost of €11.09 per operating minute, the total operating cost amounted to €665.40 per procedure. As no time differences were assumed between trocar types, operating costs were identical for reusable and disposable trocars.

### Disposal costs

The calculated disposal costs per procedure differed substantially between reusable and disposable trocars. When applying the amortised disposal costs of reusable trocars over the assumed 150 uses, the cost amounted to only €0.003 (95% CI: 0.002–0.005) per LC. This also includes the disposal costs for Bluewrap. Trocar weights used for disposal cost calculations are provided in Appendix 5. For disposable trocars, multiplying the total weight of four instruments by the hospital’s disposal cost per kilogram (SZA waste) resulted in an average disposal cost of €0.15 (95% CI: 0.09–0.22) per procedure.

### Total costs per procedure

Table 1 summarises the per-procedure costs for reusable versus disposable trocars. Disposable trocars are more expensive to purchase and incur higher disposal costs, but they require no sterilisation. Reusable trocars, in contrast, involve ongoing sterilisation expenses, while their storage and disposal costs are less than with the disposable trocars. Additionally, the purchase costs of reusable trocars are substantially cheaper. Overall, reusable trocars save €68.43 (95% CI: − 87.39–−49.08) per procedure as opposed to disposable trocars. Within the probabilistic analysis iterations, reusable trocars were less expensive than disposable trocars in 100% of iterations.

**Table 1:** Total costs per procedure

	Reusable trocars	95% CI	Disposable trocars	95% CI	Difference
Purchase costs	€8.15	5.44–11.78	€89.55	72.44–107.72	€− 81.39
Sterilisation costs	€13.31	8.37–20.29	n.a	n.a	€13.13
Storage costs	€0.08	0.04–0.13	€0.27	0.15–0.44	€− 0.19
Operation costs	€665.40	n.a	€665.40	n.a	€0.00
Disposal costs	€0.003	0.002–0.005	€0.15	0.09–0.22	€− 0.015
Total costs	€683.00	435.49–962.98	€751.43	506.69–1035.21	€− 68.80
Total annual costs MUMC + LC	€102,450.49	65,323–144,448	€112,714.71	76,004–155,281	€− 10,264.21
Total annual costs NL LC	€15,367,574.3	9,798,482.92–21,667,265.70	€16,907,206.33	11,400,606.57–23,292,232.00	€− 1,539,631.97

*CI* credible interval, *LC* laparoscopic cholecystectomy, *MUMC* + Maastricht University Medical Centre + , *n.a.* not applicable, *NL* Netherlands

Switching to reusables and applied to an annual caseload of 150 LC procedures MUMC + , this corresponds to €10,264 (95% CI: − 13,109; − 7,362) in savings per year. Nationally, assuming 22,500 LC procedures per year in the Netherlands, the potential saving is €1,539,631 (95% CI: − 1,966,386; − 1,104,355) annually for LC.

### Sensitivity analyses

#### One-way sensitivity analyses

The one-way sensitivity analyses reveal that the price of the 12 mm disposable trocar type 1 is the most influential parameter, on absolute cost differences between reusable and disposable trocars. Here, reusable trocars remained less costly than disposable trocars by €59.19 (minimum price) and €80.45 (maximum price).

The number of uses of reusable trocars also significantly impacts the results. With 50 uses (the minimum value), the total cost difference reduces to €54.88, still favouring reusable trocars. When the number of uses increases to 500 (the maximum value), the total cost difference drops further to €73.66.

Other disposable trocar prices also show a notable effect, with higher prices increasing the cost difference and favouring reusable trocars. In contrast, factors such as sterilisation wage costs, time estimates for quality control and maintenance of reusable trocars, and time estimates for decontamination and dismantling have a relatively limited impact on total costs. Total costs for reusable trocars were consistently lower than those for disposable trocars across the upper and lower limits of the most influential parameters.

**Fig. 3:**
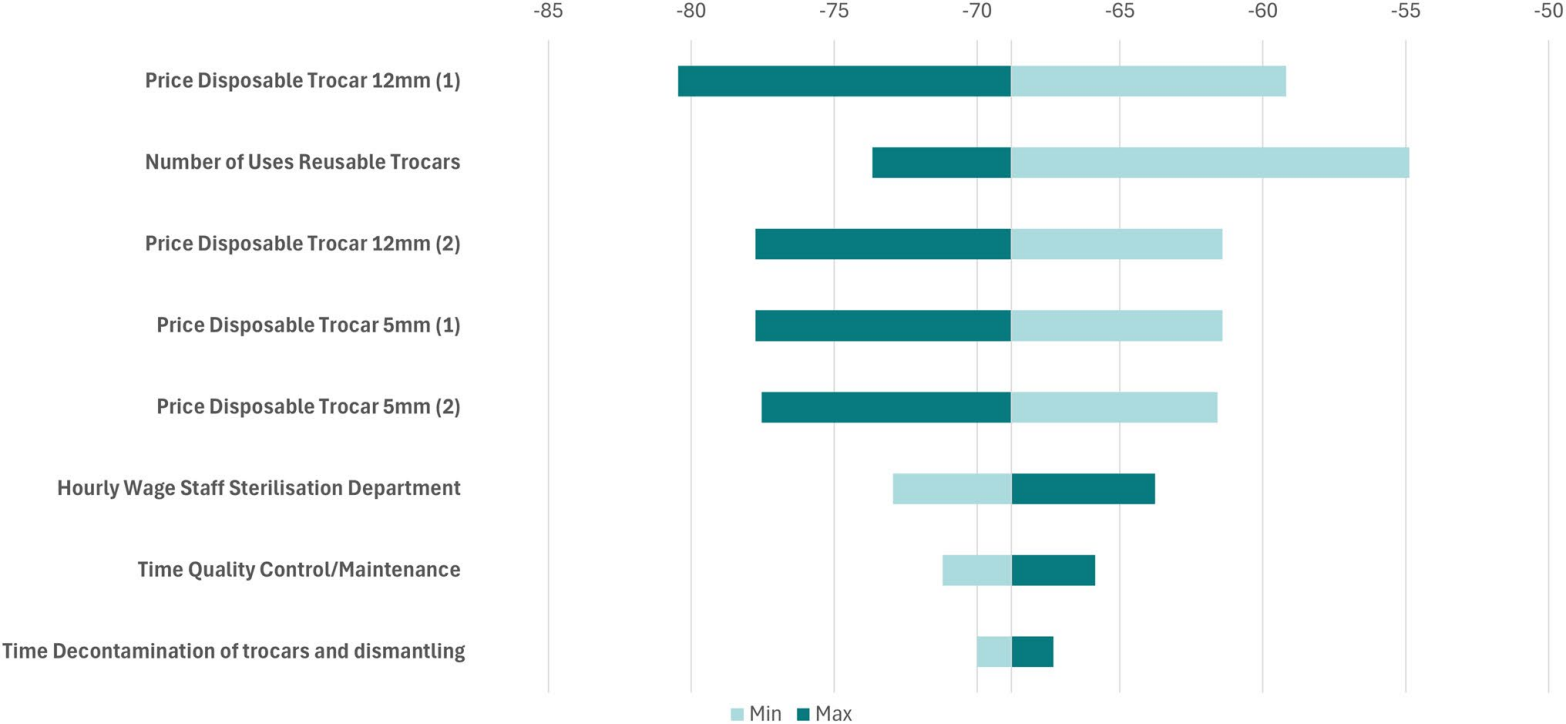
Tornado diagram one-way sensitivity analysis

Overall, the analysis indicates that the results are most sensitive to assumptions regarding reuse rates and disposable trocar costs, while other parameters contribute little uncertainty (Fig. 3).

#### Two-way sensitivity analysis

The two-way sensitivity analysis consisted of the price of the disposable trocar 12 mm type 1 and the number of uses for reusable trocars. Reusable trocars were less costly compared to disposable trocars across all combinations of varying parameters (purchase price of the disposable trocar 12 mm type 1 and number of reuses for the reusable trocars), indicating that reusable trocars are cost-saving regardless of the specific values chosen within the defined ranges for these parameters. The difference in total cost ranged from €− 44.66 to €− 84.45 per procedure. The heat diagram can be seen in Appendix 6.

### Scenario analysis

For reusable trocars, emissions of 0.236 kg CO_2_eq per procedure resulted in an additional environmental cost of €0.14, increasing the total cost to €683.14 per procedure. For disposable trocars, emissions of 1.13 kg CO_2_eq per procedure corresponded to an added environmental cost of €0.18, increasing the total cost to €751,61 per procedure.

Including environmental costs therefore resulted in a marginal increase in total procedural costs for both trocar types and did not alter the cost difference between reusable and disposable trocars.

In a scenario analysis using a mean acquisition cost for reusables of €1180 and for disposables of €162, the total cost difference was €− 141.34 with reusables being cheaper than disposables.

## Discussion

We developed a costing framework to calculate the cost of reusable and disposable trocars. The framework in this study is available as an Excel file and can be provided upon request from the corresponding author. This study shows that the use of reusable trocars for LC compared to disposables leads to cost savings for an academic hospital in the Netherlands. While cost savings per procedure are modest (€68.80), at a national level this translates into annual savings of over €1 million for LC. These findings align with previous research that found reusable surgical instruments have economic advantages [7, 19]. However, these studies did not give a complete insight into which steps are involved in the reprocessing of reusable trocars and focused on multiple instruments and not just trocars.

### Interpretation of findings

The main driver of the cost difference between the two types of trocars is the purchase price. Assuming reusable trocars are used 150 times, the cost per procedure is markedly lower for reusable trocars compared to disposable trocars. Sterilisation and maintenance costs partially offset these savings but did not negate them. Sensitivity analyses varying the purchasing price, salaries, costs of materials and equipment, and time for activities confirmed the robustness of these findings, with reusable trocars remaining the less costly option.

Including the social cost of carbon slightly favoured reusable trocars, but the cost difference was very small. These results should be interpreted with caution. Carbon emission estimates were based on a single study, and there is uncertainty around the appropriate value of the social cost of carbon [24]. Although a value endorsed by the European Commission was used, different values could change the results. Importantly, the purpose of this scenario analysis was not to demonstrate a decisive economic advantage, but to explore the potential impact of incorporating environmental externalities into procedural cost calculations. Even though the resulting difference (€0.14 per procedure) was small, it illustrates how environmental costs can be integrated into health economic evaluations and procurement decisions, consistent with WHO recommendations for climate-resilient healthcare systems.

### Strengths and limitations

A key strength of this study is its comprehensive activity-based costing approach, informed and validated by expert input and supported through process mapping. In contrast to previous research, which often omitted certain procedural steps or assumed comparable costs across activities, this analysis accounts for all relevant activities in the care process, providing a more accurate and realistic estimation of total costs [5, 8, 25–29]. The sole assumption in this study, in which no difference was expected, related to operation time. No evidence in existing literature indicates that a difference in operation time would be present. However, there are also limitations. Assumptions regarding the lifespan and maintenance of reusable trocars may not fully capture real-world variability, as premature breakage in clinical practice could influence cost outcomes. However, a conservative number of uses was assumed in the analysis, and even under one-way sensitivity analysis using a minimum of 50 uses, reusable trocars remained less expensive. It is likely that reusable trocars have a substantially longer lifespan, further reducing the purchase cost per procedure and increasing overall cost savings. One-way sensitivity analysis also showed that the overall cost difference is highly sensitive to the cost of disposable trocars. Additionally, while the social cost of carbon was included in a scenario analysis, other externalities such as water pollution, resource extraction, or toxic waste were not quantified. Further, the analysis was conducted in one hospital, which may have limits pertaining to the generalisability of results, especially to settings with different sterilisation infrastructure. Outsourcing sterilisation, for example, increases costs due to the sterilisation unit being at a larger distance, which introduces a longer logistic loop [30]. Since the total cost difference is relatively small, outsourcing sterilisation could make disposable trocars more cost-saving. We have therefore provided the detailed process map so that input is easily adapted to reflect a local context.

### Implications for policy and practice

The findings of this study have several practical implications. First, hospitals can realise immediate savings by adopting reusable trocars in LC. Secondly, although adding the environmental impact to the costs did not show a large cost difference in social cost of carbon, environmental impact is an important factor to consider when deciding which trocar to use. Third, the costing framework offers a flexible tool that other institutions can adapt to their own clinical settings, equipment lifespans, and differences in salaries to inform purchasing decisions. This framework is intended as a practical starting point for cost calculations of medical devices across other types of surgery. The freely available Excel-based tool can be readily adapted to different medical instruments and care processes, enabling systematic cost comparisons between reusable and disposable options and supporting future research in a wide range of surgical contexts.

## Conclusion

This activity-based costing analysis demonstrates that reusable trocars are a more cost-efficient option compared to disposable ones. While initial investment and sterilisation costs are higher, reusable trocars become cost-saving over time through multiple uses. Notably, this research is the first to comprehensively examine the entire cost pathway within the hospital setting. Additionally, it presents an adaptable framework that healthcare organisations can use to assess the economic implications of adopting reusable trocars, confirming that they are more economical in the long run and contribute to reduced environmental impact.

## Supplementary Information

Below is the link to the electronic supplementary material.

Supplementary file1 (DOCX 134 kb)
