# Supplementary material for: From procurement to disposal: a framework for an activity-based cost analysis of reusable and disposable trocars in laparoscopic cholecystectomy
Source: Surg Endosc. 2026 Apr 21;40(7):5838–46. doi: 10.1007/s00464-026-12827-0 (PMC13369031; doi:10.1007/s00464-026-12827-0)

**Appendix 1. Sterilisation Cost Input Parameters for One Cycle**

| **Cost Category** | **Cost Items** | **Unit** | **Cost per Unit** € | **Source** | **Year** | **Note** |
| --- | --- | --- | --- | --- | --- | --- |
| ***Materials*** | Soap 1 | ml | 0.0051 | Expert Opinion | 2025 |  |
|  | Soap 2 | ml | 0.0047 | Expert Opinion | 2025 |  |
|  | Soap 3 | ml | 0.0052 | Expert Opinion | 2025 |  |
|  | Bluewrap | Per sheet | 0.35 | Expert Opinion | 2025 |  |
| ***Equipment*** | Thermodisinfector | Per Cycle | 4.71 | Hospital Bill | 2025 | Calculation Dutch Costing Manual |
|  | Steriliser | Per Cycle | 20.53 | Hospital Bill | 2017 | Adjusted with consumer price index and Calculation Dutch Costing Manual |
| ***Utilities*** | Electricity | kWh | 0.1313 | European Wind Energy Hospital Bill | 2025 | Assumption: Electricity from whole machine is using purchased energy and not self generated |
|  | Water | m^3^ | 1.3732 | Hospital Data | 2025 | Based on the hospital bill from one month including drinkwater, VAT, fixed fees |
| ***Personnel*** | Salary Staff | h | 41 | Finance Department MUMC+ | 2025 |  |

**Appendix 2. Input Parameters (Individual Prices of Trocar Components are Confidential)**

| **Category** | **Sub-Parameter** | **Use in PSA?** | **Use in DOWSA?** | **Deterministic** | **SE** | **Distribution** | **Minimum Value** | **Maximum Value** |
| --- | --- | --- | --- | --- | --- | --- | --- | --- |
| **Operational Input Parameters** |  |  |  |  |  |  |  |  |
|  | CPI_2017 | No | No | 0,014 | 0,0028 | NA | 0 | 0 |
|  | CPI_2018 | No | No | 0,017 | 0,0034 | NA | 0 | 0 |
|  | CPI_2019 | No | No | 0,026 | 0,0052 | NA | 0 | 0 |
|  | CPI_2020 | No | No | 0,013 | 0,0026 | NA | 0 | 0 |
|  | CPI_2021 | No | No | 0,027 | 0,0054 | NA | 0 | 0 |
|  | CPI_2022 | No | No | 0,1 | 0,02 | NA | 0 | 0 |
|  | CPI_2023 | No | No | 0,038 | 0,0076 | NA | 0 | 0 |
|  | CPI_2024 | No | No | 0,033 | 0,0066 | NA | 0 | 0 |
|  | CPI_2025 | No | No | 0,035 | 0,007 | NA | 0 | 0 |
|  | Interest Rate Equipment | Yes | Yes | 0,025 | 0,005 | Gamma | 0,016178682 | 0,035710098 |
|  | Economic Life Equipment | No | No | 10 | 2 | NA | 0 | 0 |
|  | Number of Uses Reusable Trocars | Yes | Yes | 150 | 30 | Gamma | 50 | 500 |
|  | Number of Uses individually for 2 Silicone Valves, 2 Sealing Caps, 2 Cross Ventils | Yes | Yes | 90 | 18 | Gamma | 30 | 108 |
|  | Trocars used per Laparoscopic Cholecystectomy at MUMC+ | No | No | 4 | 0,8 | NA | 0 | 0 |
|  | Number of 5mm Trocars in one procedure | No | No | 2 | 0,4 | NA | 0 | 0 |
|  | Number of 12mm Trocars in one procedure | No | No | 2 | 0,4 | NA | 0 | 0 |
|  | Nets in Thermodisinfector | No | No | 12 | 2,4 | NA | 0 | 0 |
|  | Nets in Steriliser | No | No | 16 | 3,2 | NA | 0 | 0 |
|  | Trocars in one Net | No | No | 8 | 1,6 | NA | 0 | 0 |
|  | Number of Laparoscopic Cholecystectomies Netherlands per Year | No | No | 22500 | 4500 | NA | 0 | 0 |
|  | Number of LCs performed at MUMC+ annually | No | No | 150 | 30 | NA | 0 | 0 |
| **Purchasing Input Parameters** |  |  |  |  |  |  |  |  |
|  | Price Reusable Trocar 5mm | Yes | Yes | Confidential | Confidential | Gamma | Confidential | Confidential |
|  | Price 1 Pin 5mm | Yes | Yes | Confidential | Confidential | Gamma | Confidential | Confidential |
|  | Price Reusable Trocar 12mm | Yes | Yes | Confidential | Confidential | Gamma | Confidential | Confidential |
|  | Price 1 Pin 12mm | Yes | Yes | Confidential | Confidential | Gamma | Confidential | Confidential |
|  | Price 2 Sealing Caps 12mm | Yes | Yes | Confidential | Confidential | Gamma | Confidential | Confidential |
|  | Price 2 Cross Ventils 12mm | Yes | Yes | Confidential | Confidential | Gamma | Confidential | Confidential |
|  | Price 2 Silicone Valves 5mm | Yes | Yes | Confidential | Confidential | Gamma | Confidential | Confidential |
|  | Price Disposable Trocar 5mm (1) | Yes | Yes | Confidential | Confidential | Gamma | Confidential | Confidential |
|  | Price Disposable Trocar 5mm (2) | Yes | Yes | Confidential | Confidential | Gamma | Confidential | Confidential |
|  | Price Disposable Trocar 12mm (1) | Yes | Yes | Confidential | Confidential | Gamma | Confidential | Confidential |
|  | Price Disposable Trocar 12mm (2) | Yes | Yes | Confidential | Confidential | Gamma | Confidential | Confidential |
| **Storage Input Parameters** |  |  |  |  |  |  |  |  |
|  | Annual Laparoscopic Procedures MUMC+ | No | No | 3600 | 720 | NA | 0 | 0 |
|  | Storage Space 10days supply for disposable trocars | Yes | Yes | 0,24 | 0,048 | Gamma | 0,155315346 | 0,342816937 |
|  | Storage Space 10days supply for reusable trocars | Yes | Yes | 0,0792 | 0,01584 | Gamma | 0,051254064 | 0,113129589 |
|  | Price m2 Annually | Yes | Yes | 3709 | 741,8 | Gamma | 2400,269239 | 5297,950079 |
|  | Hourly Wage Staff Internal Distribution Centre | Yes | Yes | 47 | 9,4 | Gamma | 30,41592187 | 67,13498348 |
|  | Time Stock Check per month | Yes | Yes | 10 | 2 | Gamma | 6,471472739 | 14,28403904 |
| **Sterilisation Input Parameters** |  |  |  |  |  |  |  |  |
|  | Time Decontamination of trocars and dismantling | Yes | Yes | 5 | 1 | Gamma | 3,23573637 | 7,142019519 |
|  | Time Transfer of trocars into Thermodisinfector | Yes | Yes | 10 | 2 | Gamma | 6,471472739 | 14,28403904 |
|  | Time Quality Control/Maintenance | Yes | Yes | 10 | 2 | Gamma | 6,471472739 | 14,28403904 |
|  | Time Packing in Bluewrap | Yes | Yes | 1 | 0,2 | Gamma | 0,647147274 | 1,428403904 |
|  | Time Transfer of trocars into Steriliser | Yes | Yes | 10 | 2 | Gamma | 6,471472739 | 14,28403904 |
|  | Hourly Wage Staff Sterilisation Department | Yes | Yes | 41 | 8,2 | Gamma | 26,53303823 | 58,56456005 |
|  | Price Soap 1 (200l) | Yes | Yes | 1024,42 | 204,884 | Gamma | 662,9506103 | 1463,285527 |
|  | Price Soap 2 (200l) | Yes | Yes | 935,65 | 187,13 | Gamma | 605,5033468 | 1336,486113 |
|  | Price Soap 3 (200l) | Yes | Yes | 1049,36 | 209,872 | Gamma | 679,0904634 | 1498,90992 |
|  | Amount Soap 1 | No | No | 210 | 42 | NA | 0 | 0 |
|  | Amount Soap 2 | No | No | 5 | 1 | NA | 0 | 0 |
|  | Amount Soap 3 | No | No | 12 | 2,4 | NA | 0 | 0 |
|  | Number of Bluewrap Sheets per trocar set | No | No | 2 | 0,4 | NA | 0 | 0 |
|  | Price Bluewrap (100x100cm) | Yes | Yes | 840 | 168 | Gamma | 543,6037101 | 1199,859279 |
|  | Price Electricity for kWh | Yes | Yes | 0,13 | 0,026 | Gamma | 0,084129146 | 0,185692507 |
|  | Price Water per m3 | Yes | Yes | 1,37 | 0,274 | Gamma | 0,886591765 | 1,956913348 |
|  | Price Thermodisinfector | Yes | Yes | 57407,5 | 11481,5 | Gamma | 37151,10713 | 82001,0971 |
|  | Price Steriliser | Yes | Yes | 132490,0785 | 26498,0157 | Gamma | 85740,59311 | 189249,3453 |
|  | Replacement Value Thermodisinfector (V) | Yes | Yes | 57407,5 | 11481,5 | Gamma | 37151,10713 | 82001,0971 |
|  | Replacement Value Steriliser (V) | Yes | Yes | 132490,0785 | 26498,0157 | Gamma | 85740,59311 | 189249,3453 |
|  | Residual Value Thermodisinfector (R) | No | No | 0 | 0 | NA | 0 | 0 |
|  | Residual Value Steriliser (R) | No | No | 0 | 0 | NA | 0 | 0 |
|  | Maintenance Costs Thermodisinfector (5% of investment costs) | Yes | Yes | 2870,375 | 574,075 | Gamma | 1857,555356 | 4100,054855 |
|  | Maintenance Costs Steriliser (5% of investment costs) | Yes | Yes | 6624,503924 | 1324,900785 | Gamma | 4287,029655 | 9462,467265 |
|  | Annuity Factor (a,n,i) Thermodisinfector and Steriliser | No | No | 0,114258763 | 0,022851753 | NA | 0 | 0 |
|  | Annual Depreciation and Interest (k) Thermodisinfector | No | No | 6559,309947 | 1311,861989 | NA | 0 | 0 |
|  | Annual Depreciation and Interest (k) Steriliser | No | No | 15138,1525 | 3027,6305 | NA | 0 | 0 |
|  | Annual Maintenance Thermodisinfector (M) | No | No | 2870,375 | 574,075 | NA | 0 | 0 |
|  | Annual Maintenance Steriliser (M) | No | No | 6624,503924 | 1324,900785 | NA | 0 | 0 |
|  | Annual Number of Runs Thermodisinfector/Steriliser | Yes | Yes | 2132 | 426,4 | Gamma | 1379,717988 | 3045,357123 |
|  | Amount of Electricity Thermodisinfector | Yes | Yes | 1299,79 | 259,958 | Gamma | 841,1555552 | 1856,62511 |
|  | Amount of Electricity Steriliser | Yes | Yes | 2013 | 402,6 | Gamma | 1302,707462 | 2875,377058 |
|  | Amount of Water Use Thermodisinfector (combined water reverse osmosis and softened) | Yes | Yes | 166 | 33,2 | Gamma | 107,4264475 | 237,115048 |
|  | Amount of Water Use Steriliser (combined water reverse osmosis and softened) | Yes | Yes | 229 | 45,8 | Gamma | 148,1967257 | 327,104494 |
| **Operation Input Parameters** |  |  |  |  |  |  |  |  |
|  | Price Conventional OR per minute | Yes | Yes | 11,09 | 2,218 | Gamma | 7,176863268 | 15,84099929 |
|  | Operation Time Reusable Trocars | No | No | 60 | 12 | NA | 0 | 0 |
|  | Operation Time Disposable Trocars | No | No | 60 | 12 | NA | 0 | 0 |
| **Disposal Input Parameters** |  |  |  |  |  |  |  |  |
|  | Weight Reusable Trocar 5mm | No | No | 0,06 | 0,012 | NA | 0 | 0 |
|  | Weight Reusable Trocar 12mm | No | No | 0,27 | 0,054 | NA | 0 | 0 |
|  | Weight Disposable Trocar 5mm (1) | No | No | 0,04 | 0,008 | NA | 0 | 0 |
|  | Weight Disposable Trocar 5mm (2) | No | No | 0,05 | 0,01 | NA | 0 | 0 |
|  | Weight Disposable Trocar 12mm (1) | No | No | 0,05 | 0,01 | NA | 0 | 0 |
|  | Weight Disposable Trocar 12mm (2) | No | No | 0,09 | 0,018 | NA | 0 | 0 |
|  | Weight Bluewrap per trocar set (2 sheets) | No | No | 0,102 | 0,0204 | NA | 0 | 0 |
|  | Disposal cost of specific hospital waste (SZA) per kg | Yes | Yes | 0,66 | 0,132 | Gamma | 0,427117201 | 0,942746576 |
|  | Disposal cost of general waste per kg | Yes | Yes | 0,19 | 0,038 | Gamma | 0,122957982 | 0,271396742 |
| **Environmental Input Parameters** |  |  |  |  |  |  |  |  |
|  | Amount of Carbon for Reusable Trocars | No | No | 118 | 23,6 | NA | 0 | 0 |
|  | Amount of Carbon for Disposable Trocars | No | No | 565 | 113 | NA | 0 | 0 |
|  | Social Cost of Carbon per tonne | No | No | 158,39 | 31,678 | NA | 0 | 0 |

**Appendix 3. Prespecified Minimum and Maximum Value**

| **Parameter** | **Minimum Value** | **Maximum Value** |
| --- | --- | --- |
| Number of Uses Reusable Trocars | 50 | 500 |
| Number of Uses individually for 2 Silicone Valves, 2 Sealing Caps, 2 Cross Ventils | 30 | 108 |
| Price Reusable Trocar 5mm | Prespecified Value (Confidential) | Prespecified Value (Confidential) |
| Price Reusable Trocar 12mm | Prespecified Value (Confidential) | Prespecified Value (Confidential) |

**Appendix 4.. Resource Use Sterilisation**

| **Resource Use Category** | **Input Type** | **Resource Use Per Cycle** | **Source** |
| --- | --- | --- | --- |
| ***Materials*** | Soap 1 | 5ml | Manufacturer Specifications |
|  | Soap 2 | 210ml | Manufacturer Specifications |
|  | Soap 3 | 12ml | Manufacturer Specifications |
| ***Utilities*** | Thermodisinfector Electricity | 1299.7945 Wh | Manufacturer Specifications |
|  | Thermodisinfector Water | 166 l | Manufacturer Specifications |
|  | Steriliser Electricity | 2013 Wh | Manufacturer Specifications |
|  | Steriliser Water | 251 l | Manufacturer Specifications |

**Appendix 5. Resource Use Disposal**

| **Resource Use Category** | **Input Type** | **Resource Use** | **Source** |
| --- | --- | --- | --- |
| ***Weight*** | Reusable Trocar 5mm | 0.0634 kg | Manufacturer Specifications |
|  | Reusable Trocar 12mm | 0.2689 kg | Manufacturer Specifications |
|  | Disposable Trocar 5mm (1) | 0.0379 kg | Weighed by Research Team |
|  | Disposable Trocar 5mm (2) | 0.0529 kg | Weighed by Research Team |
|  | Disposable Trocar 12mm (1) | 0.0545 kg | Weighed by Research Team |
|  | Disposable Trocar 12mm (2) | 0.0907 kg | Weighed by Research Team |

**Appendix 6. Heat Diagram Two Way Sensitivity Analysis**


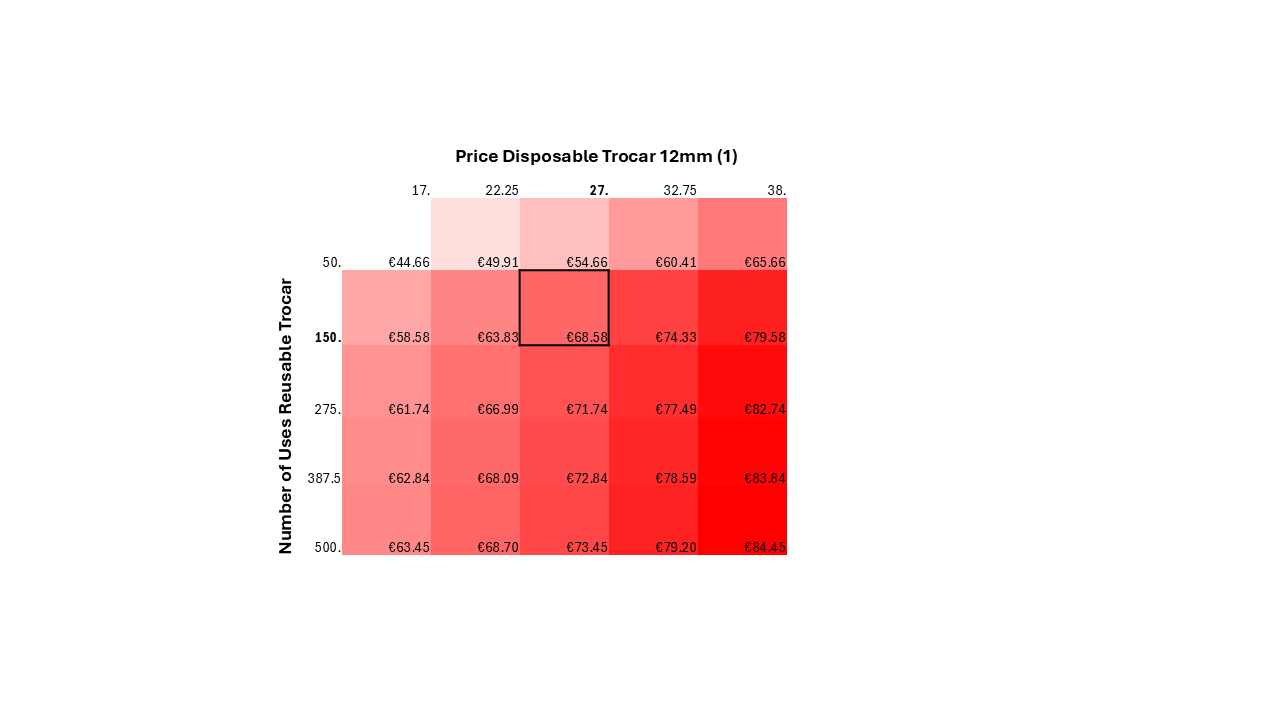

Supplement: Supplementary file 1 — Supplementary file1 (DOCX 134 kb) [file 464_2026_12827_MOESM1_ESM.docx]
